# Self-organization of synchronous activity propagation in neuronal networks driven by local excitation

**DOI:** 10.3389/fncom.2015.00069

**Published:** 2015-06-04

**Authors:** Mehdi Bayati, Alireza Valizadeh, Abdolhossein Abbassian, Sen Cheng

**Affiliations:** ^1^Mercator Research Group “Structure of Memory”, Ruhr-Universität BochumBochum, Germany; ^2^Department of Physics, Institute for Advanced Studies in Basic SciencesZanjan, Iran; ^3^School of Cognitive Sciences, Institute for Research in Fundamental SciencesTehran, Iran; ^4^School of Mathematics, Institute for Research in Fundamental SciencesTehran, Iran; ^5^Department of Psychology, Ruhr-Universität BochumBochum, Germany

**Keywords:** synfire chains, spike timing dependent plasticity (STDP), locally connected random networks, feed-forward networks, neuronal sequence

## Abstract

Many experimental and theoretical studies have suggested that the reliable propagation of synchronous neural activity is crucial for neural information processing. The propagation of synchronous firing activity in so-called synfire chains has been studied extensively in feed-forward networks of spiking neurons. However, it remains unclear how such neural activity could emerge in recurrent neuronal networks through synaptic plasticity. In this study, we investigate whether local excitation, i.e., neurons that fire at a higher frequency than the other, spontaneously active neurons in the network, can shape a network to allow for synchronous activity propagation. We use two-dimensional, locally connected and heterogeneous neuronal networks with spike-timing dependent plasticity (STDP). We find that, in our model, local excitation drives profound network changes within seconds. In the emergent network, neural activity propagates synchronously through the network. This activity originates from the site of the local excitation and propagates through the network. The synchronous activity propagation persists, even when the local excitation is removed, since it derives from the synaptic weight matrix. Importantly, once this connectivity is established it remains stable even in the presence of spontaneous activity. Our results suggest that synfire-chain-like activity can emerge in a relatively simple way in realistic neural networks by locally exciting the desired origin of the neuronal sequence.

## 1. Introduction

The propagation of neural spiking activity has been observed in various parts of the brain, including neocortex (Mao et al., 2001; Ikegaya et al., 2004; Pinto et al., 2005) and hippocampus (Miles et al., 1988; Nádasdy et al., 1999; Buhry et al., 2011; Cheng, 2013). It has been suggested that the reliable propagation and transformation of neural activity between different brain regions is crucial for neural information processing. Therefore, a number of computational and theoretical studies have studied the conditions under which neural activity can propagate reliably in neural networks (Diesmann et al., 1999; Kistler and Gerstner, 2002; Yazdanbakhsh et al., 2002; Aviel et al., 2005; Kumar et al., 2008). A prominent model for activity propagation is the synfire chain model, in which groups of neurons that fire synchronously are chained together into a larger feedforward network (Abeles et al., 1993; Mao et al., 2001; Abeles, 2009). Although isolated feedforward networks can account for the repeated dynamics in cortical networks (Diesmann et al., 1999; van Rossum et al., 2002), anatomical studies suggests biological networks are more adequately modeled by local recurrent connectivity than by feedforward structures (Hellwig, 2000).

Furthermore, most biological networks are not hard-wired; they are often structured by synaptic plasticity, such as spike-timing dependent plasticity (STDP) (Gerstner et al., 1996; Markram, 1997; Bi and Poo, 1998; Zhang et al., 1998; Kempter et al., 1999). STDP is widely thought to underlie learning processes, and is the focus of many theoretical studies (Leibold et al., 2008; Voegtlin, 2009; Masquelier et al., 2009; D'Souza et al., 2010; Gilson et al., 2010). The shape of the STDP curve has been proposed to trade off two competing features of STDP, synaptic competition and synaptic stability (Gütig et al., 2003; Morrison et al., 2007; Babadi and Abbott, 2010, 2013). In the case of anti-symmetric STDP, reversing the ordering of pre- and post-synaptic spikes reverses the direction of synaptic change. It breaks strong reciprocal connections between neuron pairs (Abbott and Nelson, 2000; Kozloski and Cecchi, 2010). Any inhomogeneity in initial synaptic weights, or in firing rates, determines which of the synapses will be potentiated and which will be eliminated (Babadi and Abbott, 2013). Through structural changes such as these, STDP also alters the network dynamics. For instance, STDP tends to enhance population synchrony in recurrent networks (Levy et al., 2001; Kitano et al., 2002a,b; Takahashi et al., 2009), and facilitates the formation of synfire chains when applied to feedforward or random networks (Hertz and Prügel-Bennett, 1996; Suri and Sejnowski, 2002).

The converse effect, that is the network activity affects the network structure, is observed as well. For instance, if some components in a network fire at a higher rate than the remaining network, the dynamics of physical (Gavrielides et al., 1998; Valizadeh et al., 2010) or biological systems (Glass and Mackey, 1988; Panfilov and Holden, 1997) change. The neurons with the highest inherent frequency can serve as pacemaker and train their post-synaptic partner neurons in networks with all-to-all (Bayati and Valizadeh, 2012) and random connections (Takahashi et al., 2009). If the neurons in a two-neuron network, which are connected mutually, fire at different intrinsic rates, STDP strengthens the synapse from the high-frequency to the low-frequency neuron and weakens the other one. This occurs if the initial synaptic strengths are set to the values which are enough for onset of frequency synchronization after changing the strengths due to the STDP. The same argument holds for larger networks as well. With discrepancy in the intrinsic firing rates of neurons, the reciprocal connections are broken in favor of the fast-to-slow links such that an effective flow of connections emerges from fast to slow neurons (Bayati and Valizadeh, 2012). This example shows how STDP as a spatially local mechanism for modification of synapses induces global structure in recurrent networks (Babadi and Abbott, 2013).

Here, we therefore asked whether local excitation together with STDP can drive the establishment of robust propagation of synchronous activity that is characteristic of synfire chains. To do so, we study the population activity in a locally connected random network (LCRN). Local excitation is provided by a small number of neurons that fire at a higher frequency (fast-spiking neurons, FSNs). We find that robust activity propagation indeed arises and does so quickly within seconds of simulated time. The synchronous activity originates at the location of the FSNs and propagates away from them. This network behavior is the result of an effective feedforward structure that emerges spontaneously in a recurrent network through STDP. Once the network reaches the steady state, synchronous activity propagation remains stable even when the local excitation is removed. It is thus conceivable that temporary local excitation is provided to a biological neural network to establish synchronous activity propagation, and to have that activity pattern continue after the excitation is removed.

## 2. Materials and methods

### 2.1. Network dynamics and topology

**Figure 1:**
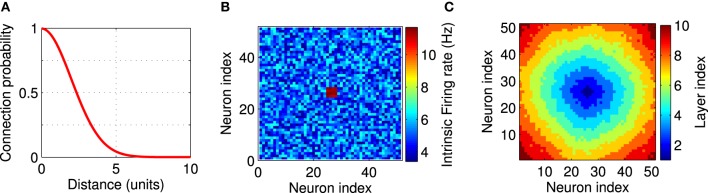
**Network setup**. The network consists of a square grid of *N* = 51 × 51 = 2601 excitatory leaky integrate-and-fire neurons. **(A)** Synaptic connectivity kernel in the two-dimensional network model. Each neuron is pre-synaptic to 40 of its neighbors. **(B)** An example for the firing rate of the neurons in the network. Each neuron receives a constant external input current, independently selected from a uniform distribution on the interval [*I*_0_ − δ, *I*_0_ + δ] with *I*_0_ = 16.21 mV, δ = 0.2 mV, which leads to background firing rates from 3.4 to 6.8 Hz. Twelve neurons in the center of the network (shown in red) receive higher input currents (*I*_0_ = 18.05 mV, δ = 0.15 mV), and therefore fire at higher rates between 11.1 and 11.7 Hz. These high-frequency neurons provide local excitation to the network above the background firing. Firing rates are measured in isolated neurons without recurrent connections. **(C)** A layer index is assigned to each neuron based on the synaptic distance to the high-frequency neurons. All neuron with the same layer index can be thought of being arranged in an abstract layer.

In our simulations we use a LCRN (Mehring et al., 2003; Kumar et al., 2008) comprising *N* = 51 × 51 units on a two-dimensional square grid. The distance between two neighboring units along the main axes of the grid is one unit. The adjacency matrix *a*_*ij*_ (Reka and Barabási, 2002) indicates whether neuron *j* projects to neuron *i* (*a*_*ij*_ = 1), or not (*a*_*ij*_ = 0). We choose the probability of a connection between any pair of neurons to decrease with the Euclidean distance (*d*_*ij*_) between the neurons since anatomical studies have found that cortical networks are mostly locally connected (Hellwig, 2000). More specifically, we connected neuron *i* to *k*_*i*_ post-synaptic partner neurons, which are chosen at random according to a local connectivity kernel, a Gaussian with zero mean and standard deviation σ = 2 (in units of the grid spacing) (Figure 1A). The post-synaptic partners were determined as follows. We sampled a random number according to the connectivity kernel, which indicates the distance between the pre- and post-synaptic neurons, and a random number from a uniform distribution between zero and 360, which indicates the direction from the pre- to the post-synaptic neuron. The connection was discarded, if the chosen position of the post-synaptic partner laid outside the boundaries of the network, which was more frequently the case for neurons at the edges of the network. We repeated the sampling of post-synaptic partners 40 times, so that *k*_*i*_ ≤ 40. Our connection procedure allows only one connection between two neurons. Anatomical studies suggest σ ≃ 0.5 mm (Hellwig, 2000). So our model covers a cortical surface of about 6 cm^2^ with only 2601 neurons. That means, we assume that the network described here constitutes a subset of a larger cortical network whose effect is considered as external currents to the modeled neurons (Kumar et al., 2008; Hahn et al., 2014).

The neurons' subthreshold dynamics were modeled by a leaky integrate-and-fire (LIF) model:

(1) $$\tau_{m}\frac{\text{d}}{\text{dt}}V_{i}(t)=V_{\text{rest}}-V_{i}(t)+I_{i}+\sum_{j}I_{ij},$$

where *V*_*i*_ represents the membrane potential of neuron *i* and *i* = 1, 2, …, *N*. The membrane potentials were set to random initial values at the beginning of each simulation. τ_*m*_ = 20 ms is the membrane time constant, *V*_rest_ = −70 mV the resting membrane potential, *I*_*i*_ the external input current and *I*_*ij*_ the synaptic current from neuron *j* to neuron *i*. Although these inputs appear as currents, they are measured in units of the membrane potential mV since a factor of the membrane resistance has been absorbed into their definition. The differential equations were solved using the Runge-Kutta second-order method with a timestep of 0.1 ms. Whenever the membrane potential of a neuron reaches a fixed threshold at *V*_*th*_ = −54 mV, the neuron generates an action potential, the membrane potential is reset to the resting potential, and a refractory period of 2 ms followed. Each time a neuron spikes, a synaptic current *g*_*ij*_ is transmitted from the pre-synaptic to the post-synaptic neuron as a pulse, with a delay of *D* = 1 ms regardless of the distance between the connected neurons. Thus, the synaptic dynamics is neglected. Writing the neuron's response function (Dayan and Abbott, 2001) as *x*_*j*_ (*t*) = ∑_*m*_ δ (*t* − *t*^*m*^_*j*_), where *t*^*m*^_*j*_ is the time of the *m*-th spike of neuron *j* and δ (*x*) is the Dirac delta function, the synaptic current *I*_*ij*_ is given by

(2) $$I_{ij}=a_{ij}\;g_{ij}\;x_{j}(t),$$

In the units that we adopted here, *g*_*ij*_ represents the synaptic strength. It is positive throughout this study since we only modeled excitatory synapses. All synaptic weights were initially set to *g*_0_ = 0.02 mV.

Inhomogeneity in the intrinsic firing rates were imposed by unequal external currents. The external input currents of all neurons, except for the FSNs, were randomly chosen from a uniform distribution on the interval [*I*_0_ − δ, *I*_0_ + δ]. With *I*_0_ = 16.21 mV and δ = 0.2 mV, the background firing rates range between 3.4 and 6.8 Hz. Local excitation was provided to the network by choosing those *n* neurons that are closest to the center of the network and providing them with higher input currents (*I*_0_ = 18.05 mV, δ = 0.15 mV). These neurons therefore fire at higher rates between 11.1 and 11.7 Hz (Figure 1B), which is why we call them FSNs. These background firing rates are measured in isolated neurons without recurrent connection. Firing rates are different when the network is recurrently connected as described above and fluctuate during the evolution of the network (see **Figure 7A**). Throughout this paper *n* = 12, unless otherwise noted.

The synaptic strengths evolved according to a pair-based STDP rule with nearest spike neighbors interaction (Izhikevich and Desai, 2003), neglecting interactions between remote spike pairs (Froemke and Dan, 2002; Pfister and Gerstner, 2006). A change of synaptic strength Δ*g*_*ij*_ was induced by a pair of pre- and post-synaptic action potentials with time difference Δ*t* = *t*_post_ − *t*_pre_. The functional relation between the synaptic modification and the pairing interval was

(3) $$\Delta g_{ij}=\left\{ \begin{array}{l} A_{+}\exp\left(-|\Delta t|/\tau_{+}\right)\text{ if }t_{post}>t_{pre}\\ -A_{-}\exp\left(-\text{|}\Delta t\text{|/}\tau_{-}\right)\text{ if }t_{post}\leq t_{pre} \end{array}\right.$$

The positive parameters *A*_+_ and *A*_−_ specify the maximum potentiation and depression, respectively. We expressed the synaptic strengths in units of the membrane potential (mV), so *A*_+_ and *A*_−_ have units of mV. The time constants τ_+_ and τ_−_ determine the temporal spread of the STDP window for potentiation and depression, which have been reported to be in the 10 - 20 ms range (Song et al., 2000). We impose hard bounds on the synaptic weights (0 < *g* < *g*_max_, where *g*_max_ = 2 g_0_), by truncating any modification that would take a synaptic weight outside the allowed range.

Therefore, all synapses in the network are excitatory, which is in accord with *in vitro* findings that synchronous activity propagation depends mainly on excitatory synaptic connections (Pinto et al., 2005), but may be modulated by inhibitory neurons (Mehring et al., 2003; Aviel et al., 2005).

To set the value of *A*_±_, we assumed that synaptic weakening through STDP is, overall, slightly larger than synaptic strengthening (*A*_−_ τ_−_ > *A*_+_ τ_+_). This condition ensures that uncorrelated pre- and post-synaptic spikes weaken synapses (Song et al., 2000). In our simulations, we used *A*_−_ τ_−_ / *A*_+_ τ_+_ ≃ 1.06. The individual parameters were *A*_+_ = 5 × 10^−5^ mV, *A*_−_ = 4.4 × 10^−5^ mV, τ_+_ = 10 ms, and τ_−_ = 12 ms.

### 2.2. Network analysis

To better illustrate the structure of the network, we assigned each neuron a layer index to indicate its distance from the FSNs (Figure 1C). We define the layer index *l*_*i*_ as the smallest number of directed edges that are necessary to move from a FSN to neuron *i*. Neurons with the same layer index are considered to be in the same abstract layer (Masuda and Kori, 2007). In Figure 1C, all pixels of the same color represent neurons in the same layer. In a strictly feedforward network, neurons would project only to other neurons that are located in a higher layer. To compare the network structures that emerge after synaptic plasticity to a feedforward network, we therefore quantified the connectivity between the abstract layers in our recurrent networks.

In reference to the FSNs, for each layer (*l*), we call the sum of the weights of all the *ij* links for which the layer index of their post-synaptic neuron is larger than the pre-synaptic one (*l*_*i*_ > *l*_*j*_) as “forward synaptic flow”:

(4) $$C_{l}^{+}=\sum_{l_{j}\;=\;l,\;l_{i}>l_{j}}g_{ij},$$

And the sum of the weights of all the *ij* links for which the layer index of their post-synaptic neuron is smaller than the pre-synaptic one (*l*_*i*_ < *l*_*j*_), as “backward synaptic flow”:

(5) $$C_{l}^{-}=\sum_{l_{i}\;=\;l,\;l_{i}<l_{j}}g_{ij},$$

To quantify the asymmetry of connections, we introduced the difference between the forward and backward synaptic flows for each layer *C*_*l*_ = *C*^+^_*l*_ − *C*^−^_*l*_ as the feedforward parameter. For simplicity, we normalized the feedforward parameter by dividing it by its total forward and backward synaptic flows. A positive feedforward parameter means that the forward synapses are stronger than the backward synapses. The opposite would be indicated by a negative feedforward parameter. Note that in the calculation of feedforward parameter only the inter-layer connections are considered and the large value of feedforward parameter does not rule out the presence of intra-layer connections.

To quantify the effective imbalance in the network we introduced an average feedforward parameter

(6) $$C_{\text{net}}=\frac{1}{N_{l}}\sum_{l}C_{l},$$

in which *N*_*l*_ is the total number of abstract layers. The average feedforward parameter can be used as a tool to survey the evolution of network and determine the emergence of feedforward structures (Bayati and Valizadeh, 2012). When all the feedforward parameters are zero the average feedforward parameter is also zero, but the inverse is not true.

### 2.3. Analysis of network activity

**Figure 2:**
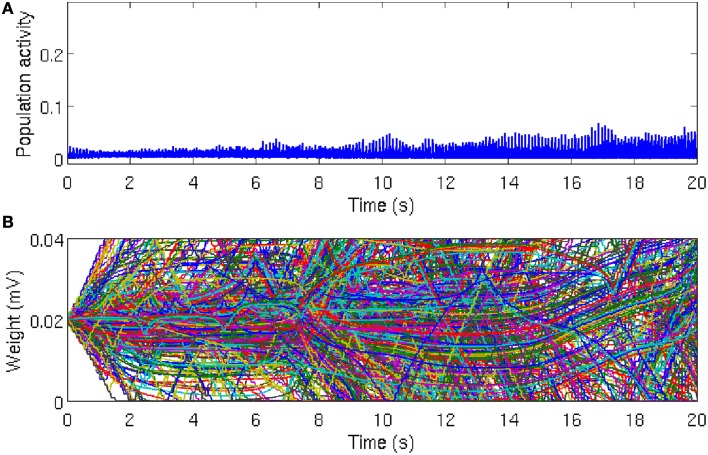
**Population activity remains asynchronous in the absence of local excitation**. **(A)** Global network activity, as measured by the average number of neurons firing within a time bin of 1 ms, increases over time due to ongoing synaptic plasticity. However, the network activity remains relatively low and noisy, which indicates that the neurons in the network are firing asynchronously. While there is a tendency for the peak of the population activity to increase slightly, reflecting more synchronous activity, there is no evidence for global synchrony in the network. **(B)** Time course of 500 randomly chosen synaptic weights. Most of the synaptic weights fluctuate between the bounding values, which are *g* = 0 mV and *g* = 0.04 mV, respectively. Since STDP eliminates synaptic loops in neural networks, after a sufficiently long simulation time, about 50% of the synaptic weights reach the threshold values, but there is no specific direction for the elimination of synaptic connections. The reason is that the heterogeneity between the firing rates are large enough and the initial synaptic weights are not strong enough to overcome the inhomogeneity (disorder) in the network and synaptic connections are eliminated in a random directions in the network.

**Figure 3:**
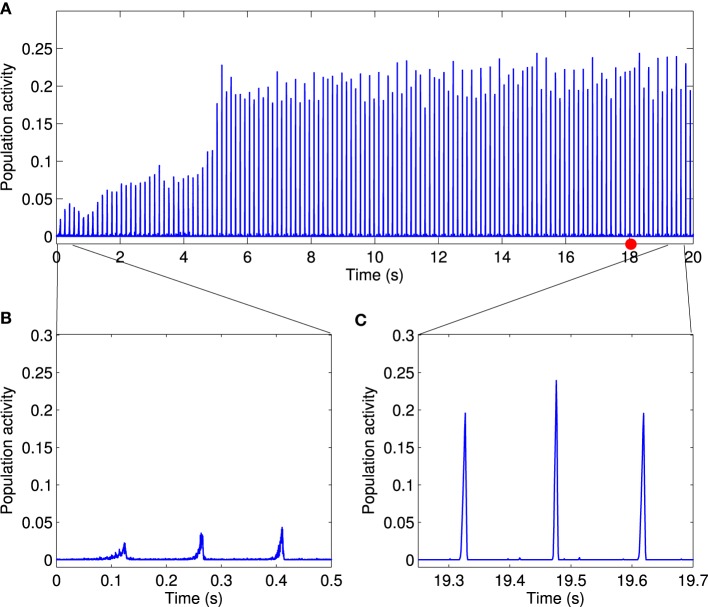
**Global synchronous activity is established by local excitation**. **(A)** In presence of local excitation, after about 5 s simulation time, global synchrony emerges in the network. Periodic network activity with relatively large amplitudes indicates that the system is in a one-cluster state. Insets of the network activity at magnified time scale show that **(B)** in the transient state synchronized network activity emerges gradually and **(C)** activity peaks are precise in the steady state. Thus, local excitation enables the network to overcome the disorder in the firing rate of neurons and to establish global synchronous activity. We used one particular population burst [shown by red circle in **(A)**] to show reliable synchronous activity propagation in **Figures 4A,B**.

To study the coherence of the neural activity in the network, we used the average response of all the neurons in the network

(7) $$X(t)=\frac{1}{N}\sum_{i}x_{i}(t),$$

which we call the population activity. In our simulation, the population activity was calculated in time bins of 1 ms. Asynchronous firing of neurons results in low and noisy population activity (Figure 2A). By contrast, large values of the population activity indicates that the network is active coherently, such as during oscillatory behavior (Figure 3). The population activity can thus be used to measure the degree of synchrony in the network.

In this study, we were interested in the temporal fine structure of population bursts in the network activity and therefore we identified the time periods in which the population bursts occurred with the following algorithm. We first defined a search window of 180 ms, which corresponds roughly to the average period of neurons in the network. This search window was moved through time in steps of 15 ms until the population was silent at the beginning of the search window *t*_0_, i.e., *X*(*t*_0_) = 0. Once this condition was satisfied, we similarly moved the end of the search window *t*_1_ forward in time until *X*(*t*_1_) = 0. Therefore, the width of the search window was variable. If at any time between *t*_0_ and *t*_1_, the population activity exceeded the threshold *X*_*th*_ = 0.015, we considered the search window to contain a population burst and used it for further analysis (see next paragraph). Otherwise, we rejected the search window.

To quantify how orderly neural activity propagates through the abstract layers, we calculated the rank-order correlation between the time of the first spikes *f*_*i*_ that the neurons fired within the search window and the layer indices *l*_*i*_. We refer to this correlation value as the propagation parameter.

(8) $$\rho =\frac{\sum_{i}\left(x_{i}-\bar{x}\right)(y_{i}-\bar{y})}{\sqrt{\sum_{i}{\left(x_{i}-\bar{x}\right)}^{2}}\sqrt{\sum_{i}{\left(y_{i}-\bar{y}\right)}^{2}}},$$

where *x*_*i*_ is the rank of neuron *i* in the list *f*_*i*_, and *y*_*i*_ is its rank according to *l*_*i*_. The means of these values are represented by *x* and *y*, respectively.

## 3. Results

### 3.1. Activity in a locally connected, random network is asynchronous despite STDP

As the asynchronous state is a more realistic description of ongoing cortical activity in the absence of strong external excitation, we set the initial conditions such that the network activity remains asynchronous. In this case, the population activity remains relatively low and noisy with irregular small peaks due to the synchronous firing of few neurons by chance (Figure 2A). In line with this irregular activity, the synaptic weights in the network fluctuate, as evident in the evolution of 500 randomly selected synaptic weights (Figure 2B). It is well-known that after a long time, most of the synapses reach the limiting values imposed by the hard bounds in the conventional linear STDP (Song et al., 2000). However, in the intermediate time range no specific network structure emerges and most of the synaptic weights remain in-between the bounding values. These results demonstrates that in the homogeneous network, in the absence of local excitation, STDP cannot establish synchronous firing. Similar asynchronous network activity has been reported for a different type of STDP rule (Morrison et al., 2007).

### 3.2. Synchronous activity propagation develops due to local excitation

It was reported that the presence of neurons with higher firing rate can substantially change the dynamics of neural networks (Masuda and Kori, 2007; Bayati and Valizadeh, 2012). In our simulation, we introduced stronger inputs to *n* = 12 neurons than to the other neurons in the network (see Materials and Methods). This setup can be related to a biological neural network in which only a small subset of neurons receive external inputs while the remaining neurons do not. With the local excitation, the network dynamics gradually changes from asynchronous initially to synchronous after about 5 s simulated time (Figure 3). The periodic peaks in the population activity in the stationary state is related to the maximum firing rate of the background firing rate (6.8 Hz). We confirmed that an overall increase in network activity, e.g., by increasing the background firing rate of all neurons to the range [7.4 − 10.4] Hz, does not give rise to synchronous activity propagation (data are not shown). We therefore conclude that local excitation in a LCRN with STDP drives the network toward global synchronous activity.

**Figure 4:**
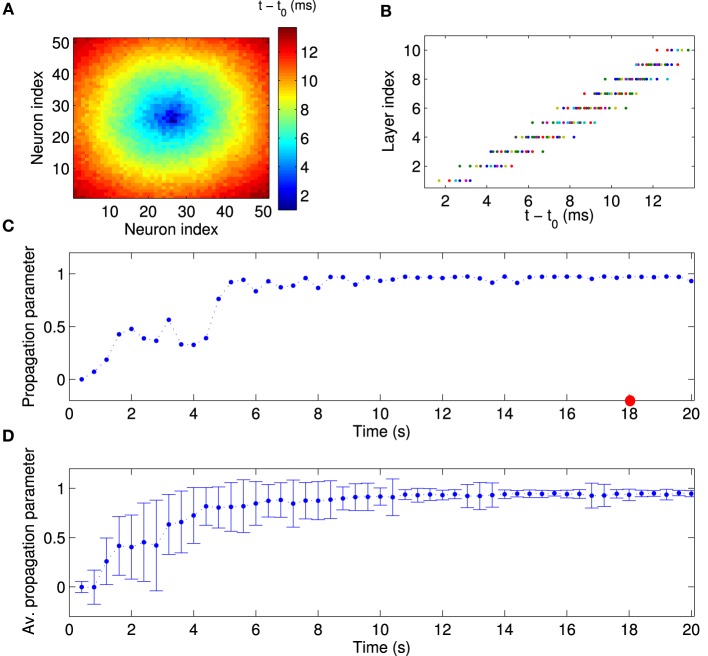
**Quantitative analysis of synchronous activity propagation**. **(A)** One particular population burst of activity propagation in the stationary state. Colors indicate the spike time of each neuron relative to the beginning of the population burst (*t*_0_ = 18 s). Note how the activity propagates from the first layer (high-frequency neurons) to higher layers (compare to Figure 1C). **(B)** Direct comparison of spike time and assigned layer index for each neuron, for the same time window as in **(A)**. Neurons indeed fire sequentially according to their layer index. Since neurons in the same layer also fire synchronously, activity in our network propagates much like in synfire chains. Note that this synchronous propagation emerged in our network in a self-organized manner and was not hand-tuned. To quantitatively measure synchronous activity propagation in the network, we calculated the rank-order correlation between layer index and spike time (propagation parameter). For instance, the propagation parameter in this example is about 0.98 (red circle in **C**). **(C)** The propagation parameter as a function of time for one example network. In this case, activity propagation is established after about 5 s. The fluctuations are reduced after 10 s. **(D)** The propagation parameter averaged across 50 simulations with different random input currents. Errorbars indicate the standard deviation. It decreases as the synchronous propagation in the network stabilizes after about 10 s.

Next, we examined the population activity within a population burst at a finer temporal resolution to reveal the relative spike timing of the neurons. Figure 4A shows one particular population burst in which the neural activity propagates from the center outward. A comparison with the layer index (Figure 1C) suggests that neural activity starts in the FSNs (first layer) and then propagates to downstream layers. In addition, neurons that belong to the same or nearest layers fire synchronously. These observations are confirmed by a direct comparison of relative spike times during the population burst and the assigned layer index (Figure 4B). The population burst in Figures 4A,B shows that activity is propagated along the abstract layers much like in synfire chains (Abeles, 1991, 2009). Note that this synchronous activity is evident in the first layer, in that spike times in the first layer have a narrow distribution (Figure 4B), and then propagates through the network. This pattern contrasts with other models in which, for instance, synchrony builds up as activity propagates through the layers of a feedforward network (Tetzlaff et al., 2002, 2003). Our model therefore shows how synfire chains could emerge in recurrent networks driven by a small number of FSNs.

To quantitatively measure synchronous activity propagation in the network, we calculated the propagation parameter for each population burst, i.e., the rank-order correlation between layer index and time of first spike (see Materials and Methods). For instance, the propagation parameter for the example in Figures 4A,B is about 0.98, which indicates highly reliable activity propagation. We then followed the propagation parameter across time. During the initial stages of the simulation, the propagation parameter increases rapidly, then fluctuates at a high level, and finally stabilizes after about 10 s (Figure 4C). This temporal pattern is seen consistently across multiple simulations, when the propagation parameter is averaged across 50 simulations with different random input currents (Figure 4D). Note, in particular, the higher standard deviation for the data points during the transient state. When the network reaches the stationary state, the variability is fairly small. During the transient state, the network is in two or more cluster states and the propagation can be seen in part of the network (results not shown). In conclusion, local excitation drives network changes such that initially asynchronous firing becomes coherent and neurons in each layer fire successively one after another.

### 3.3. Local excitation and STDP create feedforward network structures

**Figure 5:**
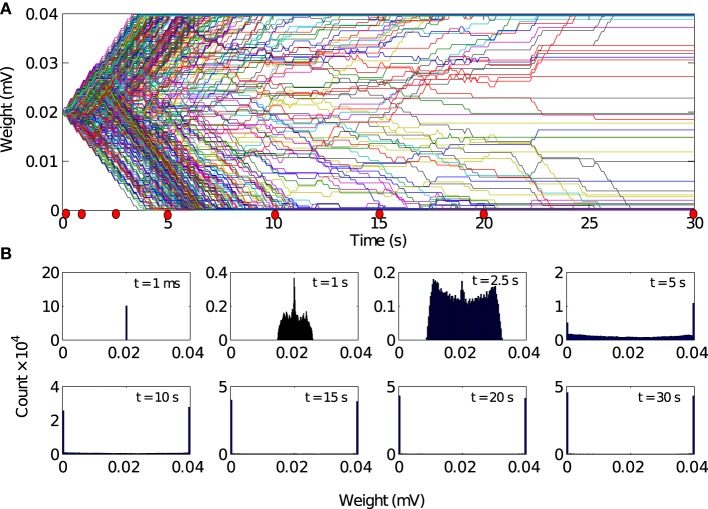
**Evolution of synaptic weights driven by local excitation**. **(A)** Results are shown for 500 randomly selected synapses. Most weights (about 90%) converged toward the limiting values of 0 mV and 0.04 mV. **(B)** Histogram of the synaptic weight distribution at the different timepoints marked by red dots in A. The distribution of synaptic weights converge from delta-function distribution to a stable bimodal distribution. There are no apparent changes in the weight distributions between *t* = 20 s and *t* = 30 s. So we conclude that the network has reached a steady state after 20 s of simulation time.

How does local excitation lead to the emergence of synchronous activity propagation? We hypothesized that the answer lies in the changes in the network structure, rather than the dynamic effects of FSNs alone. The evolution of 500 randomly selected synaptic weights reveals that the network activity and STDP force most of the synaptic weights (about 90%) to converge to the bounding values (Figure 5A), *g* = 0 mV and *g* = 0.04 mV. We sought to confirm this observation by examining the distribution of all synaptic weights at different times during the simulation. After the transient state, the synaptic weights quickly reach a stable bimodal distribution (Figure 5B). Note that the convergence of the synaptic weights to the limiting values is much faster than in the case of the networks without local excitation.

**Figure 6:**
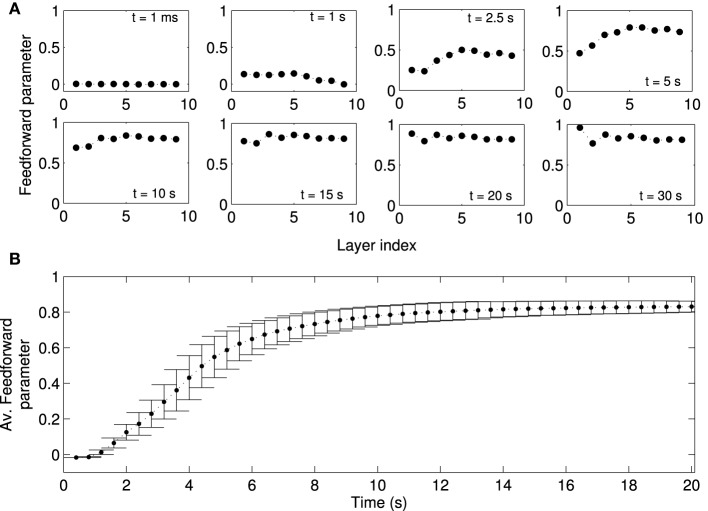
**Formation of a feed-forward structure in locally connected networks**. Local excitation drives STDP in the network such that synapses from lower to higher layers become strengthened, while the synapses in the reverse direction are weakened. Note that “layers” in our study refers to abstract layers based on synaptic distance to high frequency neurons (Figure 1C). We do not manually set up a feedforward structure in our network. **(A)** The feedforward parameter as a function of the layer is shown at different times (same as Figure 5B) for one representative network simulation. Initially, the feed-forward parameters are close to zero because the initial synaptic weights are symmetric. Values of the feedforward parameter near 1 indicate a feedforward structure. Due to plasticity the feedforward structure emerges gradually in the network, first in higher layers (*t* = 5 s) and later in the lower layers. **(B)** Feedforward parameter as a function of simulation time averaged across 50 simulations with different random input currents. There are no apparent changes in the structure of the network after *t* = 20 s. Errorbars show the standard deviation (SD) at each timepoint. Note that the SD decreases as the weights reach the steady state.

We next studied the directionality of synaptic connections, which is more important for activity propagation than the distribution of synaptic weights. Due to the random assignment of initial synaptic connections, the synaptic projections in the forward and backward directions are initially balanced, i.e., the initial feedforward parameters in all layers are close to zero (Figure 6A). Over time, a feedforward structure gradually emerges in the network. The feedforward structure first emerges in the layers with larger indexes, i.e., those far from the FSNs, and later in layers close to the FSNs. After about 20 s simulation time, the feedforward parameter is close to one in all the layers.

The example studied above is representative of other instances of the random network. When averaged across 50 simulations, in which networks receive a different sampling of random input currents, the evolution of the average feedforward parameter clearly shows the three stages discussed for the example above. The network is initially (*t* < 2 s) symmetric and the average feedforward parameter is near zero with little variability (Figure 6B). As the network structure changes in the transient state (2 s < *t* < 8 s), the average feedforward parameter increases and there is large variability. When the network reaches the stationary state, the average feedforward parameter approaches the maximal value and the variability is markedly reduced. The evolution of the network structure, while not perfectly matching, parallels the evolution of network activity.

### 3.4. Synfire chain activity persists in absence of local excitation

**Figure 7:**
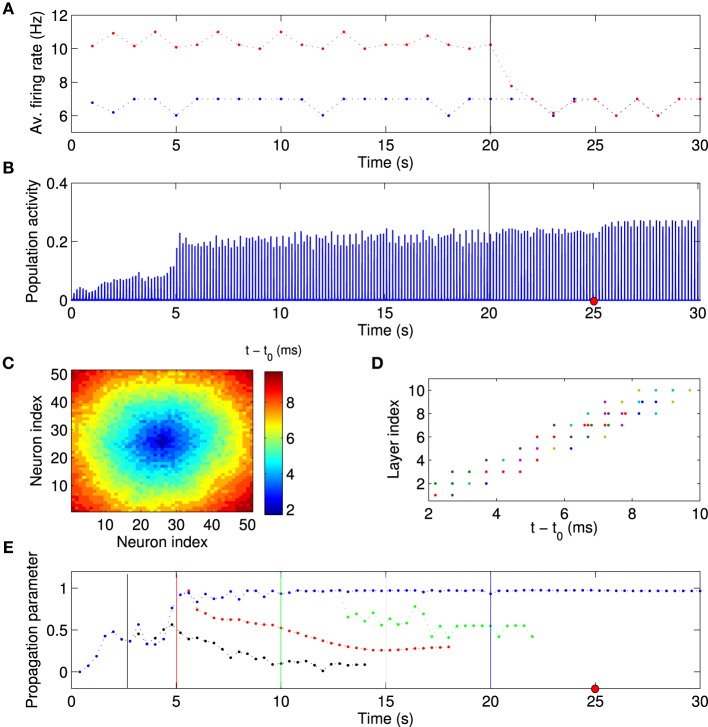
**Synchronous activity propagation persists after local excitation is removed**. **(A)** Average firing rate of background neurons (blue points) and high-frequency neurons (red points) as a function of time. After the network has reached the stationary state, the local excitation is removed at *t* = 20 s (vertical line), i.e., the high-frequency neurons receive an input current from the same distribution as the other neurons in the network. Despite this manipulation, **(B)** the network activity remains highly synchronized and the activity propagation remains stable **(C–E)**. **(C,D)** Example of spike times, taken at the time indicated by the red dot in **(B)**. **(E)** If anything, the activity propagation appears to be more stable since there are smaller fluctuations in the propagation parameter (blue data points). The same analysis was performed with the local excitation removed at different times as indicated by the vertical lines (black, red, green, and blue). When the local excitation is removed at earlier time points, activity propagation destabilizes. This simulation used the same network parameters as the one shown in Figure 6. Visual inspection suggests that the feedforward parameter (Figure 6A) determines whether activity propagation remains stable after the local excitation has been removed. If the network structure is feedforward, including the first layer, i.e., the feedforward parameter is larger than about 0.75, then stable synfire-chain- like propagation remains stable after learning.

Since we view the local excitation as a model of some salient, but temporary, external stimulation (Ritz and Sejnowski, 1997), one might expect that the firing rates of the FSNs return to baseline once the external stimuli are removed. We therefore asked whether the established network structure and activity patterns remain stable after the local excitation ceases. After the network has reached the stationary state (*t* = 20 s), we reset the external input current to the FSNs to values drawn from the same distribution as for the background neurons. Thus, the firing rates of the former FSNs are now the same as those of the other neurons in the network (Figure 7A). Importantly, the population activity remains synchronous (Figure 7B) and neural activity continues to propagate through the abstract layers of the network (Figures 7C–E). Surprisingly, the values of the population activity and propagation parameter are slightly higher and less variable after local excitation has been removed (Figures 7B,E), indicating more stable activity propagation.

**Figure 8:**
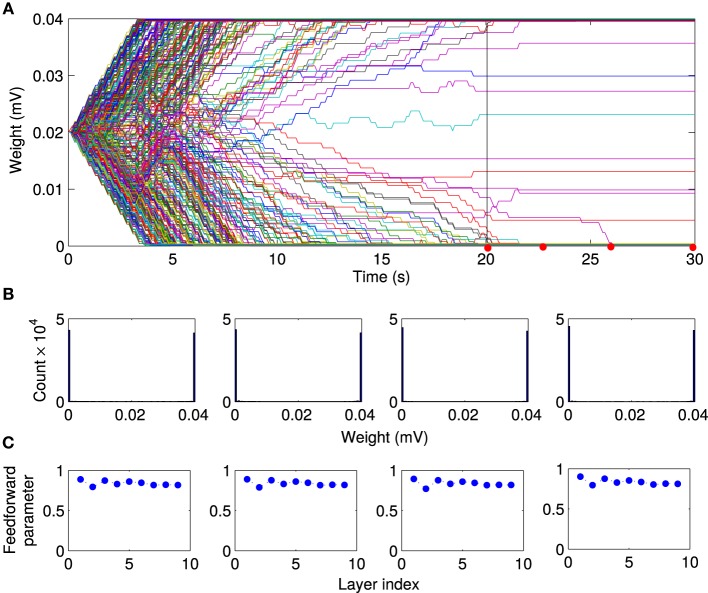
**Stability of feed-forward structure after removing local excitation**. Comparing the evolution of the network structure in the presence of local excitation *t* < 20 s and after removing it (*t* > 20 s). **(A)** After the local excitation is removed, the synaptic weights remain at their stationary values, although STDP is still active in the network. Furthermore, as shown at selected timepoints [red dots in **(A)**], **(B)** the bimodal distribution of synaptic weights, and **(C)** the feedforward structure remains stable after removing the local excitation.

We then investigated the mechanism responsible for the synchronous activity propagation through the network in more detail in two different ways. First, we removed the local excitation at earlier timepoints (*t* = 2.5 s, *t* = 5 s, and *t* = 10 s) in the same network we used to generate Figure 6. Synchronous activity propagation degraded in each of these cases. By comparing the network structure at these times (Figure 6A) and the propagation parameter after removing the local excitation (Figure 7E), we found that only when the whole network, including the first layer, reaches a feedforward structure, the synfire-chain-like propagation remains stable without local excitation. This result is consistent with previous studies of synfire chains, but unlike in previous models, STDP continues to operate in our model and could therefore change synaptic weights after the local excitation has been removed. In other words, the overall network structure is stable against changes in individual synapses, which continue to occur in our model (Figure 8).

**Figure 9:**
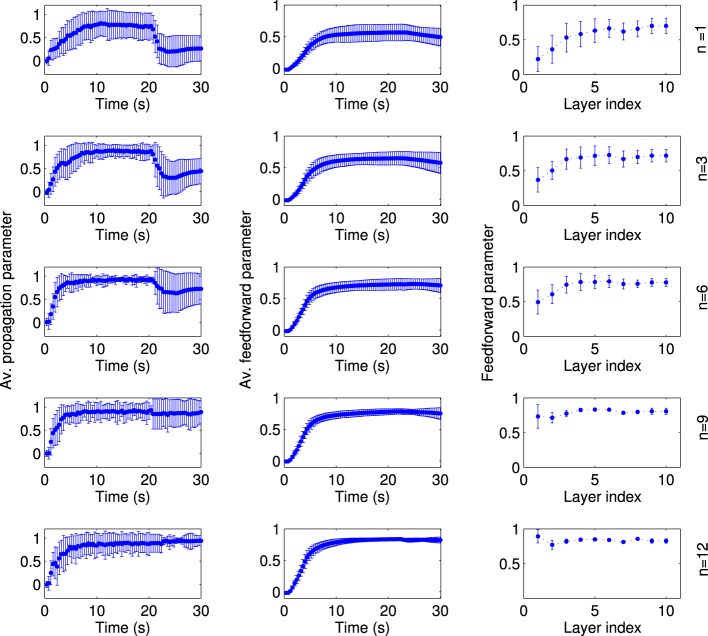
**The extend of local excitation that is required to establish lasting network structures**. Each row shows the results for local excitation involving a different number *n* of fast spiking neurons, as indicated on the far right. The networks are simulated in the presence of local excitation for 20 s, after which the local excitation is removed. All data points are averaged across 50 simulations with different random input currents. The average propagation parameter (left column) shows that stable activity propagation can be established for any number of fast-spiking cells. However, after the local excitation is removed the average propagation parameter remains stable only for *n* ≥ 8. The reason for this differentiation is evident in the network structure. For *n* < 8, the average feedforward parameter (middle column) is smaller and, importantly, the feedforward parameter of the fist layer at the stationary state (right column) is smaller as compared to the simulations with *n* ≥ 8.

Second, we changed the extent of the local excitation, i.e., the number of FSNs, while fixing the average firing rate of FSNs at *f*_*FSN*_ ≈ 11.4 Hz. Otherwise, networks simulation were setup as before and run for *t* = 20 s, before the local excitation was switched off. It is striking, but consistent with experimental evidence (Miles and Wong, 1983), that even a single FSN can drive synfire chain activity in the network (Figure 9, top left panel). However, the network activity pattern is not stable when local excitation is removed (*t* > 20 s). This stability occurs only for larger numbers of FSNs, *n* > 8 (Figure 9, left column). The reason for this difference appears to be that the structure of the network is not completely feedforward for small number of FSNs (Figure 9, middle column). In particular, at the end of the learning period (*t* = 20 s) the feedforward parameter of the first layer is quite small for *n* ≤ 8 (Figure 9, right column). These results suggest that even a small number of FSNs can change the network structure for synchronous activity propagation, but only a larger number of FSNs can drive more substantial changes in the network that are sustained after the FSNs are removed.

**Figure 10:**
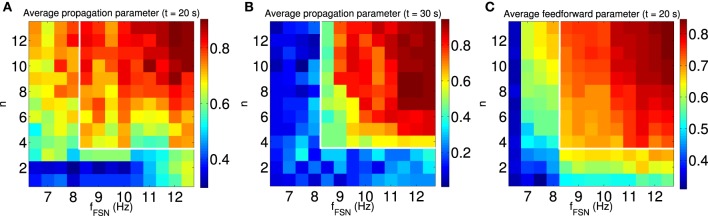
**Synchronous activity propagation is sustained only if the network is feedforward**. We systematically characterized the propagation of synchronous activity in the network as a function of two properties of the local excitation: the extend and the intensity of local excitation. **(A,B)** The average propagation parameter in the steady state [*t* = 20 s, **(A)**] and after removing the local excitation [*t* = 30 s, **(B)**] indicated by the color scale. The white outline indicates the region with sustained propagation of synchronous activity in the absence of local excitation. Note that for some parameters synchronous activity propagates through the network only when the local excitation is present. **(C)** The average feedforward parameter in the steady state (*t* = 20 s) is indicated by color scale. Only when the structure of the network is mostly feedforward (average feedforward parameter approximately ≥ 0.7), is the synchronous activity propagation sustained after removing the local excitation (see white outlines). All data points are averaged across 100 simulations with different random input currents.

To confirm the relationship between feedforward network structure and sustained synchronous activity propagation, we systematically study the dependence of these properties on the model parameters. Since plasticity is driven by the FSNs, we chose as independent variables the extent of the local excitation, as introduced above, and the intensity of local excitation, i.e., the difference between the average firing rate of FSNs and the background firing rate. In the presence of local excitation, synchronous activity propagation emerges in the steady state (*t* = 20 s; Figure 10A) for a large number of parameter combinations. However, in only a subset of these cases is synchronous activity propagation sustained after the local excitation is removed (*t* = 30 s; Figure 10B, see white outlines). In the same parameter regime (Figure 10, white outlines), and only there, the average feedforward parameter is approximately ≥ 0.7 (Figure 10C). These results confirm our hypothesis that the synchronous activity propagation is sustainable in the absence of local excitation if the network has a strong feedforward structure.

## 4. Discussion

Here, we have explored the dynamics of the network activity and the structure of a locally connected random network (LCRN) using computational simulations. Specifically, we have studied the role of local excitation in shaping such a network through spike-timing dependent plasticity (STDP) to generate synchronous activity propagation through the network. This synfire-chain like pattern of activity emerges when a feedforward structure self-organizes in the network.

Our work is clearly distinct from previous models of synchronous activity propagation in neural networks. Most previous models use hard-wired synaptic connections (Abeles, 1991; Diesmann et al., 1999; Kumar et al., 2010; Azizi et al., 2013, for instance), but models in which synchronous activity propagation self-organizes also differ from ours. For instance, Fukai and colleagues (Kitano et al., 2002a,b) discuss models in which a chain of a handful of clusters emerged. The neurons within the clusters fire nearly synchronously, while the clusters excite each other sequentially. Thus, in their model, activity propagates in volleys through the network. By contrast, in our model, activity propagates at the single neuron level and there is significant overlap between the activity in different layers, which appears to be a better description of *in vivo* data (Ikegaya et al., 2004).

### 4.1. Relation to other studies and predictions

Our modeling results can account for modeling findings which show that network activity propagation can be transiently synchronized by external stimulation and return to an asynchronous state after the stimulation is removed (Tsodyks and Sejnowski, 1995; Mehring et al., 2003). We showed here that synchronous activity propagation remains transient, if the network structure has not become fully feedforward during the stimulation, because the external stimulation was not long-lasting, extensive or intense enough (Figures 7, 10). We therefore predict that changing the stimulation parameters in the experiments will result in synchronous activity propagation that persists beyond the duration of stimulation. Furthermore, our results predict that the switch between transient and stable synfire chain activity is controlled by the degree to which the network structure is feedforward. Finally, our model predicts that the feedforward network structure starts to forms first in the last layer and then propagates backwards to the first layer. These predictions of our model can be contrasted with an alternative account of the experimental observation based on balanced excitation and inhibition (Marder and Buonomano, 2004). In this account, the network initially responds with an explosion in activity when the stimulation is applied, and then switches to an asynchronous state due to the global inhibition when the excitation is removed.

In our study, local excitation drives the self-organization of a largely feedforward network structure in LCRN through STDP. Our results thus further strengthen the view that feedforward network structures are particularly well-suited to propagate synchronous activity. An *in vitro* study reported that the neural activity becomes progressively more synchronized as it propagates through the layers of constructed feedforward networks (Reyes, 2003). Furthermore, after removing local excitation in the stationary state (*t* > 20 s), the synchronous activity propagation appears to be robust. For instance, when we resampled the external input to all neurons from the interval of background firing rates ([3.4–6.8] Hz), the network continued to support the propagation of synchronous activity (data not shown). This result suggests that the feedforward structure of the network can compensate for the perturbation in the network inputs. The robustness to other forms of network manipulation remains to be explored in the future.

Although neural synchronization might be important for information processing in the nervous system, excessive synchrony may impair brain function and causes several neurological disorders (Pyragas et al., 2007). Therefore, it is important for the brain to control this spontaneous synchronous activity. In Section 3.4, we showed that synchronous activity persists even though local excitation is switched off. We also found that reducing the average background firing rate by 0.6 Hz switches off the synchronous activity propagation (data not shown). In other words, the activity in the network is driven by the external input and synchronized by the synaptic connections. Without any external input the activity in the network ceases.

### 4.2. The importance of the initial weights and baseline firing rates

The population activity is sensitive to any sufficiently large inhomogeneity. In the absence of local excitation the network breaks into several synchronous subclusters, probably because of small disparity in the firing rates. For a range of parameters, local excitation can override this clustering effect and establish a feedforward network structure. However, after the local excitation is removed, the network structure breaks into the subclusters again and abolishes the synfire chain activity. To avoid this clustering effect, we had to adjust the initial weight matrix and dispersion of the input currents. If the initial weights are low and baseline firing rates are drawn from a wide distribution, the neurons near the boundary cannot be trained by the central ones and network activity remains asynchronous. On the other hand, some neurons randomly have higher firing rate than their neighboring neurons and due to that they are able to entrain some of their neighbors. As a result, there is a tendency of the population activity to become more synchronous and increasing in peak of population activity (Figure 2A), despite the absence of local excitation.

The asynchronous network state is considered a more realistic model of cortical background activity in the absence of external stimulation to the network (Brunel, 2000; Mehring et al., 2003). However, the initial weights cannot be too small since activity has to propagate through the abstract layers in order to train the weights (Kumar et al., 2008, 2010; Jahnke et al., 2013). Therefore, the initial weights and dispersion of input currents in our model were chosen to balance these opposing requirements.

There is little experimental data to suggest what the distribution of initial synaptic weights before the learning period look like, even though the initial weights play a central role in the dynamics of the network activity and structure. For instance, Babadi and Abbott (2013) have shown for networks of two excitatory neurons that different initial weights of the reciprocal connections lead to different final network structures in the steady state. The exact relationship depends on the STDP rule and on the firing rates of the two neurons. If the STDP rule is dominated by depression, which is the case in our model, and firing rates are the same, then the stronger weight will prevail and the other connection is eliminated. That is, unless the weights are both weak, in which case both synapses are eliminated. If, on the other hand, the two neurons have different firing rates and equal initial synaptic weights, the low frequency neuron receives more excitation, and as a result, the synapse from the high to low frequency neuron grows and the other synapse weakens, ultimately leading to a feedforward network (Bayati and Valizadeh, 2012, for instance). These results also suggest that in large networks, the initial distribution of synaptic weights might be an important factor in determining the final steady state structure of the network. In our current simulations, we used identical weights; future work is needed to study the impact of the initial weight distribution on the synchronous activity propagation.

### 4.3. Other directions for future studies

In addition to the initial synaptic weights and baseline firing rates, a number of other variables might affect synchronous activity propagation. The network dynamics of a simulated recurrent network of spiking neurons where all connections between excitatory neurons are subject to STDP is quite sensitive to the particular STDP-rule that is used. Variants of this STDP rule have been proposed in order to prevent the instability induced by conventional STDP (Pfister and Gerstner, 2006; Babadi and Abbott, 2010, 2013; Clopath and Gerstner, 2010; Clopath et al., 2010). In this study, we used the additive STDP model which modifies the synapses independently of the synaptic weight (Song et al., 2000). This STDP rule leads to a bimodal synaptic weight distribution, which in turn forms synfire-chain-like structure in our work. In contrast, weight-dependent STDP does not lead to the emergence of synfire-chain-like connectivity patterns from a random architecture, despite local excitation of selected neuron groups (Morrison et al., 2007). The main reason is that weight-dependent STDP gives rise to a unimodal weight distribution and strong synapses are harder to potentiate than weak ones. While the network effects of the STDP rule can be partly predicted by the results for a two-neuron network, our work shows that the emergent structure is also affected by the initial network architecture and the presence of local excitation. Further studies are needed to reveal how different STDP rules affect the emergent structure.

Another variable of interest are synaptic delays which can be implemented either as axonal delay or dendritic delay. Previous studies showed that these different type of delays lead to different results. When only dendritic, and not axonal, delays are implemented (Morrison et al., 2007), simultaneous firing of two reciprocally connected neurons lead to the selective strengthening of recurrent connections between those neurons, instead of exerting a decoupling force as observed in the case of implementing the axonal delay (Lubenov and Siapas, 2008). Here we have used an axonal delay of 1 ms for all synapses regardless of the distance between the neurons. This might be another reason for the discrepancy between our results and those of Morrison et al. (2007).

On the other hand, several studies have suggested that conduction delays are heterogeneous (Soleng et al., 2003; Pyka et al., 2014) and that this heterogeneity has important functional consequences (Izhikevich, 2006; Pyka and Cheng, 2014; Sadeghi and Valizadeh, 2014). It was shown previously that neuronal synchrony can arise in a network which contains long conduction delays (Vicente et al., 2008). Nevertheless, more work is needed to study whether the results reported here can be reproduced in networks with heterogeneous and long conduction delays. There is an overwhelming consensus in neuroscience that information processing in the brain is organized in functional modules (Perkel and Bullock, 1968) and that these modules are further arranged in hierarchies, such as in the visual system (Payne and Peters, 2001). If we consider LCRNs to be the modules, in which activity is synchronized locally, as we show in this paper, then an interesting question is how a system with multiple synaptically linked modules would behave.

## 5. Conclusion

Synfire chains have been popular for modeling the synchronous propagation of neural activity in neural networks. Here we found a biologically plausible mechanism for the self-organization of synfire-chain-like activity by combining local excitation and STDP in a LCRN. Local excitation could be supplied either by external stimuli or by another brain region. Only a few seconds of local excitation suffice to drive the establishment of a feedforward structure that persists even after the local excitation is removed.

## Author contributions

Conceived and designed the experiments: MB, AV, AA, and SC. Performed the experiments: MB and SC. Analyzed the data: MB, AV, AA, and SC. Contributed reagents/materials/analysis tools: MB. Wrote the paper: MB and SC.

### Conflict of interest statement

The authors declare that the research was conducted in the absence of any commercial or financial relationships that could be construed as a potential conflict of interest.
